# Transcriptomic crossroads: Decoding genes and pathways that connect alopecia areata with chronic inflammatory skin disorders

**DOI:** 10.1016/j.xjidi.2026.100487

**Published:** 2026-05-08

**Authors:** Hadis Abdolahzadeh, Sabrina Henne, Amir Mokhlesi, Jörg Wenzel, Stefanie Heilmann-Heimbach, Regina C. Betz, F. Buket Basmanav

**Affiliations:** 1 Institute of Human Genetics, School of Medicine, University Hospital Bonn, University of Bonn, Bonn, Germany; 2 Department of Animal Sciences and Marine Biology, Faculty of Life Sciences and Biotechnology, Shahid Beheshti University, Tehran, Iran; 3 Department of Dermatology and Allergy, University Hospital Bonn, Bonn, Germany

**Keywords:** Alopecia areata, Atopic dermatitis, Gene expression, Psoriasis, Vitiligo

## Abstract

Alopecia areata (AA) is an immune-mediated hair loss disorder that frequently co-occurs with chronic inflammatory skin disorders, such as atopic dermatitis, vitiligo, and psoriasis. Emerging evidence suggest that such comorbidity profiles represent clinical AA subtypes with distinct etiological underpinnings. However, mechanistic insights remain limited owing to a lack of molecular datasets from comorbid patient cohorts. In this study, we addressed this gap by leveraging publicly available case-control gene expression datasets from AA, atopic dermatitis, vitiligo, and psoriasis for an integrative analysis. Our results revealed a statistically significant overlap between gene expression signatures of AA and each chronic inflammatory skin disorder, suggesting that their co-occurrence is not coincidental. Downstream analyses of shared gene expression signatures suggested catecholamine signaling and hair/skin-pigmentation–related processes as possible drivers of AA and vitiligo codevelopment, whereas skin barrier defects and dysregulation of specific immune response pathways may underlie the comorbid development of AA with atopic dermatitis or psoriasis. This study provides a foundation for future mechanistic investigations into skin, hair follicle, and immune system interactions that drive distinct comorbid AA subtypes.

## Graphical abstract

**Figure:**
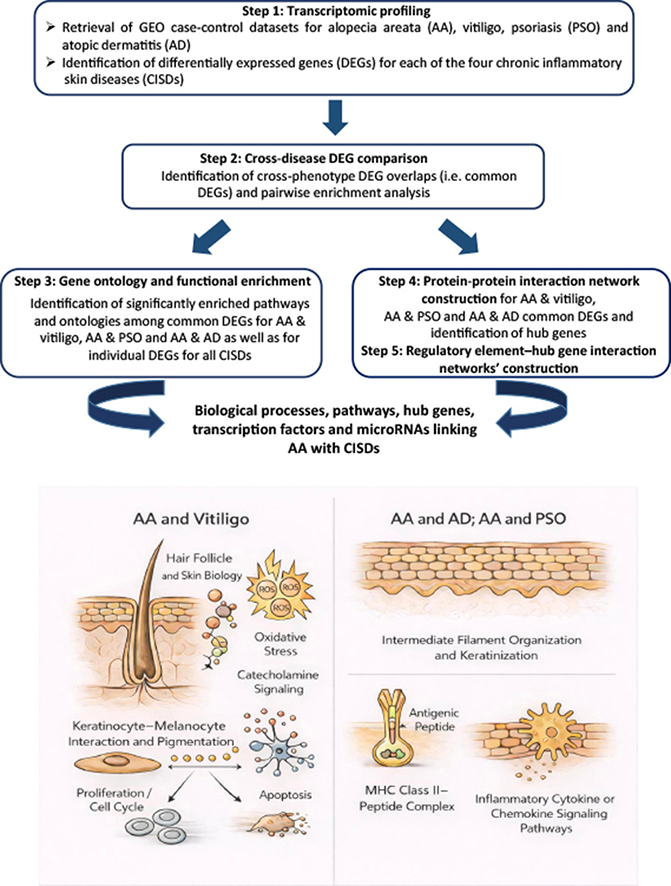

## Introduction

Alopecia areata (AA) is a common autoimmune hair loss disorder (lifetime risk of ∼2%), affecting individuals of all ages and both sexes (Pratt et al, 2017; Villasante Fricke and Miteva, 2015). Its clinical course is highly variable and often unpredictable. Some patients experience a single episode of hair loss followed by spontaneous remission, whereas others may suffer persistent hair loss without recovery. However, in most cases, the disease follows a relapsing-remitting course characterized by recurrent episodes that vary in severity and duration (Lintzeri et al, 2022; Tosti, 2023). The extent of hair loss can range from a single well-defined patch to multiple patches and, in severe cases, may progress to complete loss of scalp hair (alopecia totalis) or total body hair (alopecia universalis).

Epidemiological studies have shown a strong clinical association between AA and several chronic inflammatory skin disorders (CISDs), particularly atopic dermatitis (AD), vitiligo, and psoriasis (PSO) (Holmes et al, 2023; Kridin et al, 2020; Lee et al, 2019; Ly et al, 2023; Mohan and Silverberg, 2015). Recently, Friedrich et al (2025) have shown in a large Central European cohort that 26.7, 4.6, and 2.7% of patients with AA self-reported comorbid AD, vitiligo, or PSO, respectively (Friedrich et al, 2025). Importantly, the study indicated that patients with AA with comorbid AD are at significantly increased risk for early-onset, severe, and prolonged hair loss in comparison with patients with AA without any immune-mediated comorbidities. In addition, comorbid vitiligo was significantly associated with a greater likelihood of prolonged hair loss, whereas comorbid PSO appeared to confer a protective effect, being significantly associated with a reduced risk of prolonged hair loss (Friedrich et al, 2025).

These findings, together with evidence from other studies (Goh et al, 2006; Kageyama et al, 2021), suggest that comorbid presentations of AA may represent clinically distinct subtypes, each with potentially unique etiological and pathobiological underpinnings. However, progress in understanding these mechanisms has been hampered by a lack of molecular and cellular data from patient cohorts stratified by comorbidity profile.

In this study, we address this knowledge gap through a comparative analysis of publicly available case-control gene expression datasets from AA, AD, PSO, and vitiligo. This integrative approach allowed us to identify shared molecular signatures, shedding light on the biological pathways that may drive the comorbid development of AA with specific CISDs. Our findings provide a foundation for a more refined molecular classification of AA subtypes and offer direction for future mechanistic investigations.

## Results

**Figure 1:**
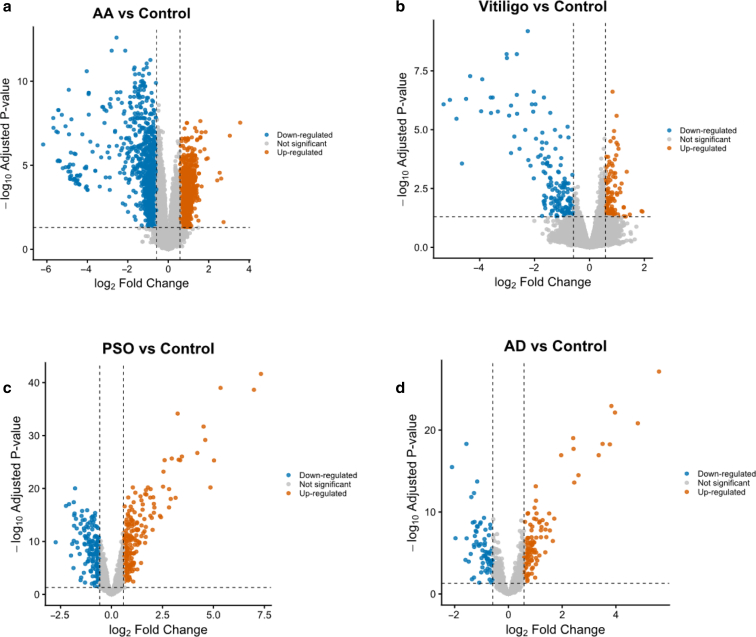
**Identification of individual DEGs in AA, vitiligo, PSO, and AD.** Volcano plots for (**a**) AA, (**b**) vitiligo, (**c**) PSO, and (**d**) AD showing up and downregulated genes in lesional skin biopsies of cases versus healthy controls. AA, alopecia areata; AD, atopic dermatitis; DEG, differentially expressed gene; PSO, psoriasis.

**Figure 2:**
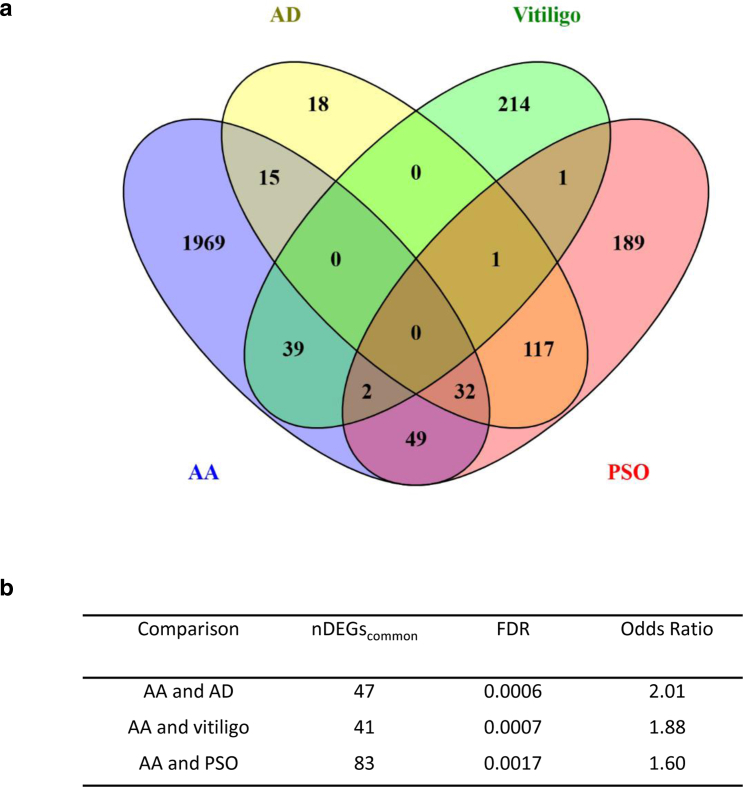
**Evaluation of molecular signature sharing across the 4 phenotypes.** (**a**) Number of overlapping DEGs across the 4 phenotypes presented by a Venn diagram. (**b**) Enrichment test to determine whether the observed DEG overlap between AA and vitiligo, PSO, or AD is statistically significant. *P*-values were corrected for multiple testing using the Benjamini–Hochberg FDR. Enrichment results for DEG overlaps across vitiligo, PSO, and AD are presented in Supplementary Table S1. AA, alopecia areata; AD, atopic dermatitis; DEG, differentially expressed gene; FDR, false discovery rate; PSO, psoriasis.

In total, 2106, 257, 391, and 183 individual differentially expressed genes (DEGs) were identified in AA, vitiligo, PSO, and AD, respectively (Figure 1). We identified no DEG that overlapped across all phenotypes (Figure 2a). Among the 35 DEGs shared across 3 phenotypes, 32 were common to AA, AD, and PSO; whereas 2 DEGs were common to AA, vitiligo, and PSO; and 1 was common to AD, vitiligo, and PSO (Figure 2a). Enrichment analysis for phenotype pairs revealed significant DEG overlap between AA and each CISD, with the strongest overlap observed with AD (false discovery rate = 0.0006, OR = 2.01) followed by vitiligo (false discovery rate = 0.0007, OR = 1.88) and PSO (false discovery rate = 0.0017, OR = 1.60) (Figure 2b and Supplementary Table S1), suggesting that these comorbidities were noncoincidental. AD and PSO also exhibited a significant overlap of DEGs, whereas vitiligo showed no significant DEG overlap with either AD or PSO (Supplementary Table S1).

**Figure 3:**
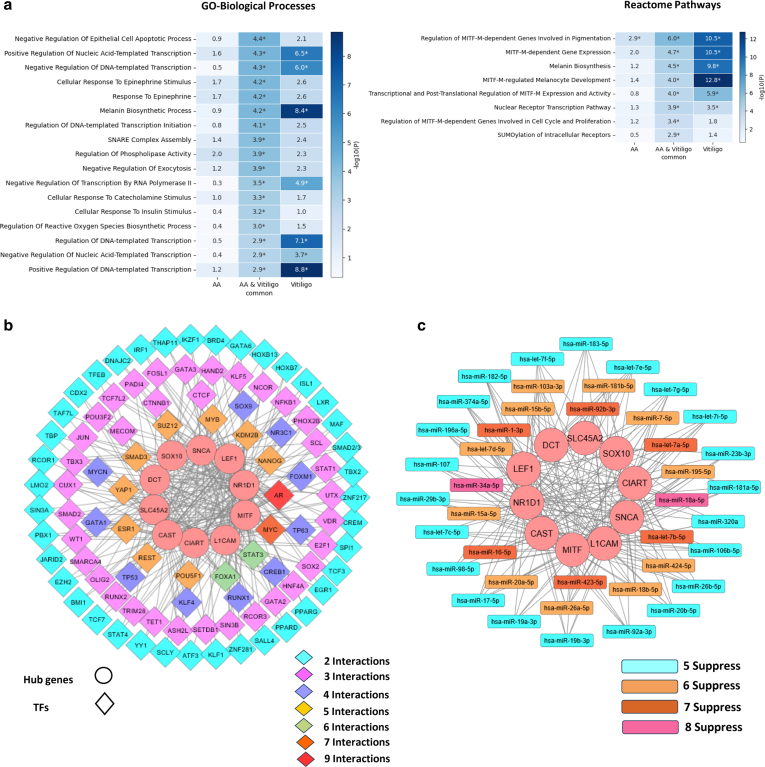
**Pathways, biological processes, and regulatory networks linking AA and vitiligo.** (**a**) Gene set enrichment analyses of AA individual DEGs, vitiligo individual DEGs, and common DEGs thereof are presented in the left, right, and middle columns, respectively. Only those pathways/biological processes that show an FDR-corrected statistically significant enrichment for the common DEGs are presented, as ranked in a top to bottom manner. (**b**) AA_vitiligo hub genes identified on the basis of the PPI networks of common DEGs for AA_vitiligo and interactions of these with TFs; (**c**) miRNAs that inhibit AA_vitiligo hub genes are presented according to the number of hub genes they each regulate. AA, alopecia areata; DEG, differentially expressed gene; FDR, false discovery rate; miRNA, microRNA; PPI, protein–protein interaction; TF, transcription factor.

**Figure 4:**
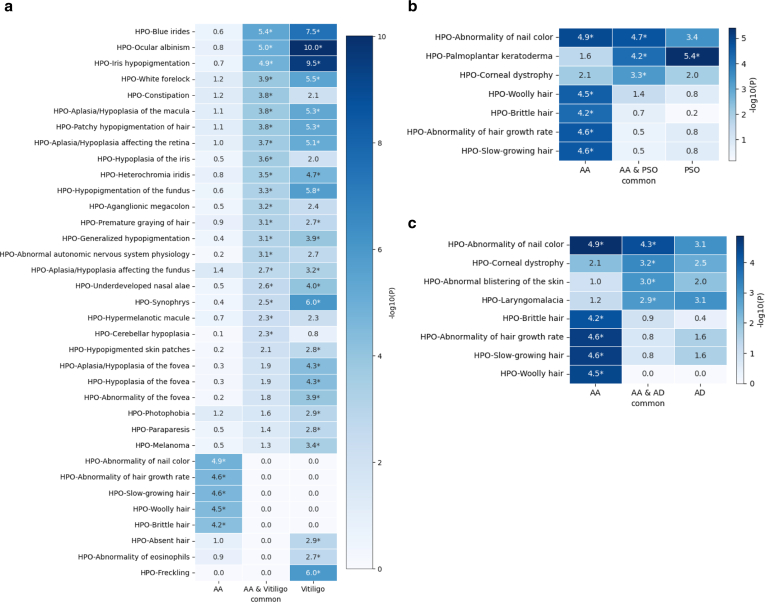
**Human phenotype ontology analysis.** Human phenotype ontology analyses of individual DEGs for AA and individual DEGs for the 3 selected comorbid CISDs as well as all common DEGs thereof are presented in the left, right, and middle columns, respectively. (**a**) AA_Vitiligo, (**b**) AA_PSO, and (**c**) AA_AD. AA, alopecia areata; AD, atopic dermatitis; CISD, chronic inflammatory skin disorder; DEG, differentially expressed gene; PSO, psoriasis;

Common DEGs for AA_vitiligo were significantly enriched for several biological processes/pathways that included negative regulation of epithelial cell apoptotic process; cellular response to catecholamine, epinephrine, or insulin stimulus; SNARE complex assembly; negative regulation of exocytosis; regulation of ROS biosynthetic process; regulation of MITF-M–dependent genes involved in cell cycle and proliferation; and SUMOylation of intracellular receptors (Figure 3a). Notably, no significant enrichment of these processes/pathways was found for the individual DEGs of either AA or vitiligo (Figure 3a). Human phenotype ontology analyses (Figure 4) revealed that AA_vitiligo common DEGs as well as individual DEGs for vitiligo were also enriched for human phenotype ontologies related to hair color/pigmentation, such as blue irides, ocular albinism, iris hypopigmentation, white forelock, and patchy hypopigmentation of hair (Figure 4a). Individual DEGs for AA, on the other hand, showed significant enrichments for human phenotype ontologies related to hair structure and growth, such as abnormality of hair growth rate, slow growing hair, woolly hair, and brittle hair (Figure 4a). Ten hub genes determined according to the protein–protein interaction (PPI) mapping of AA_vitiligo common DEGs included *LEF1*, *MITF*, *NR1D1*, *CAST*, *SNCA*, *DCT*, *L1CAM*, *CIART*, *SLC45A2*, and *SOX10* (Figure 3b), and the most connected transcription factors (TFs) to these were AR, MYC, and signal transducer and activator of transcription 3 (STAT3), each regulating the expression of 9, 7, and 6 AA_vitiligo hub genes, respectively (Figure 3b). The most highly connected microRNAs, on the other hand, were hsa-miR-18a-5p and hsa-miR-34a-5p (Figure 3c), each regulating the expression of 8 AA_vitiligo hub genes.

**Figure 5:**
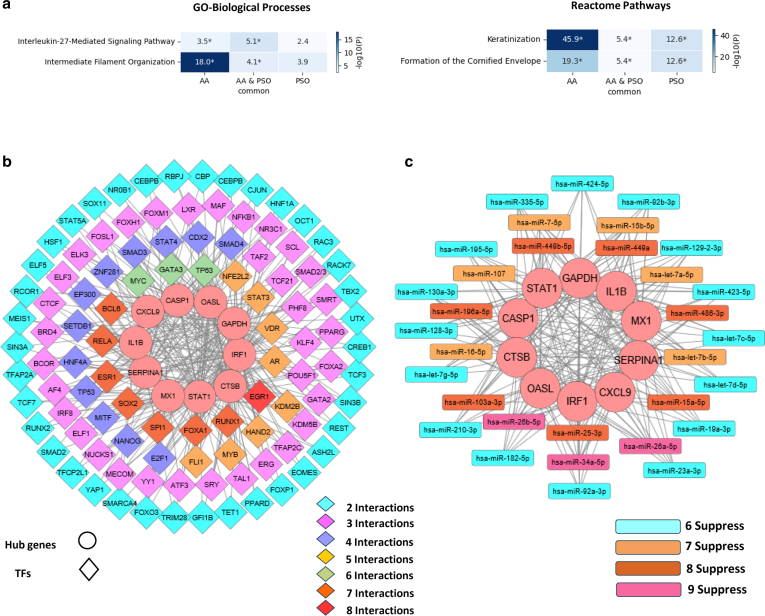
**Pathways, biological processes, and regulatory networks linking AA and PSO.** (**a**) Gene set enrichment analyses of AA individual DEGs, PSO individual DEGs, and common DEGs thereof are presented in the left, right, and middle columns, respectively. Only those pathways/biological processes that show an FDR-corrected statistically significant enrichment for the common DEGs are presented, as ranked in a top to bottom manner. (**b**) AA_PSO hub genes identified on the basis of the PPI networks of common DEGs for AA_PSO and interactions of these with TFs. (**c**) miRNAs that inhibit AA_PSO hub genes are presented according to the number of hub genes that they each regulate. AA, alopecia areata; DEG, differentially expressed gene; FDR, false discovery rate; miRNA, microRNA; PPI, protein–protein interaction; PSO, psoriasis; TF, transcription factor.

Common DEGs for AA_PSO were enriched for the IL-27–mediated signaling pathway, intermediate filament organization, keratinization, and formation of the cornified envelope (Figure 5a). Individual AA or PSO DEGs also showed significant enrichments for all of these processes/pathways. The top 10 hub genes retrieved from the PPI mapping of AA_PSO common DEGs included *CTSB*, *STAT1*, *SERPINA1*, *IL1B*, *CXCL9*, *CASP1*, *CTSB*, *GAPDH*, *MX1*, and *OASL* (Figure 5b). The top regulatory elements showing the highest interactions with these were EGR1 as a TF regulating the expression of 8 AA_PSO hub genes (Figure 5b) as well as hsa-miR-26a-5p, has-miR-26b-5p, and hsa-miR-34a-5p as microRNAs regulating the expression of 9 AA_PSO hub genes (Figure 5c).

**Figure 6:**
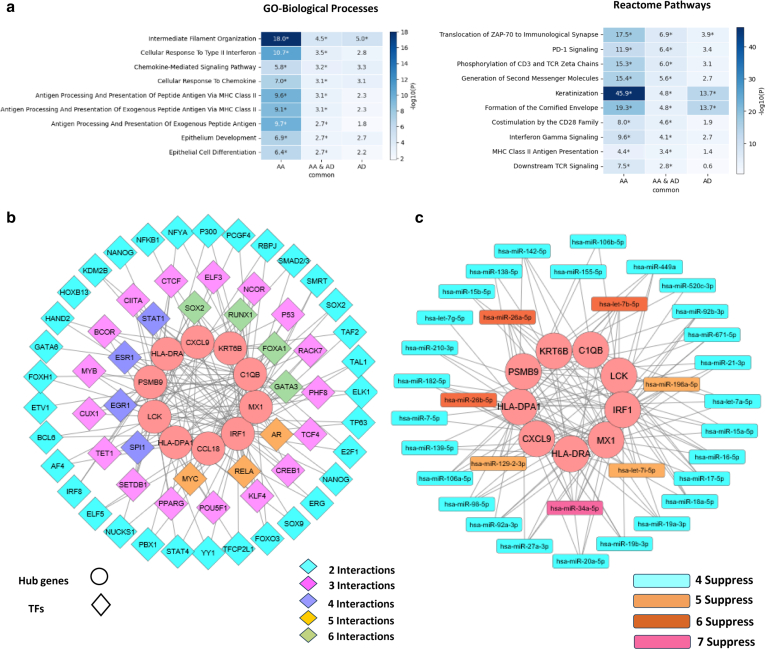
**Pathways, biological processes, and regulatory networks linking AA and AD.** (**a**) Gene set enrichment analyses of AA individual DEGs, AD individual DEGs, and common DEGs thereof are presented in the left, right, and middle columns, respectively. Only those pathways/biological processes that show an FDR-corrected statistically significant enrichment for the common DEGs are presented, as ranked in a top to bottom manner. (**b**) AA_AD hub genes identified on the basis of the PPI networks of common DEGs for AA_AD and interactions of these with TFs. (**c**) miRNAs that inhibit AA_AD hub genes are presented according to the number of hub genes that they each regulate. Of the 10 identified hub genes, 9 were included in the interaction network, because 1 gene was not recognized by the database used for miRNA interaction network construction. AA, alopecia areata; AD, atopic dermatitis; DEG, differentially expressed gene; FDR, false discovery rate; miRNA, microRNA; PPI, protein–protein interaction; TF, transcription factor.

Common DEGs for AA_AD were significantly enriched in pathways/biological processes related to the immune response and skin biology, including intermediate filament organization, epithelium development, cellular response to type II IFN, antigen processing and presentation through major histocompatibility complex class II molecules, translocation of ZAP-70 to immunological synapse, and PD-1 signaling (Figure 6a). However, individual DEGS for AA also showed significant enrichments for all of these annotations. On the other hand, intermediate filament organization, translocation of ZAP-70 to immunological synapse, and keratinization were also significantly enriched among individual AD DEGs as well (Figure 6a). The 10 top hub genes identified on the basis of the PPI mapping of AA_AD common DEGs included *IRF1*, *MX1*, *HLA-DPA1*, *PSMB9*, *HLA-DRA*, *K6B*, *LCK*, *CCL18*, *CXCL9*, and *C1QB* (Figure 6b), whereas SOX2, RUNX1, FOXA1, GATA3, and has-miR-34a-5p were the top regulatory elements demonstrating the highest number of interactions with these hub genes (Figure 6b and c).

## Discussion

In this study, we performed a comparative transcriptomic analysis and downstream investigation of DEGs shared between AA and other CISDs, including AD, vitiligo, and PSO, with the goal of identifying converging molecular signatures and biological pathways that may underlie the comorbid manifestation of AA with these distinct conditions. Our results revealed a distinct pattern indicating a stronger convergence on end-tissue–related factors (ie, cellular processes related to skin and hair follicle biology) in the AA_vitiligo constellation compared with the AA_AD and AA_PSO pairs. This was particularly evident given that no classic immune-response–related annotations were significantly enriched among the AA_vitiligo common DEGs or hub genes identified from their PPI networks. In contrast, many of the significantly enriched annotations as well as hub genes derived from the AA_AD and AA_PSO common DEGs were associated with immune-related processes.

Among the AA_vitiligo common DEGs, several interesting significantly enriched annotations emerged that were related to each other. Four of these corresponded to catecholamine signaling, regulation of ROS biosynthetic process, regulation of epithelial cell apoptotic process, and regulation of MITF-M–dependent genes involved in cell cycle and proliferation. Catecholamines are implicated in the pathobiology of both AA and vitiligo through 2 key mechanisms: they can induce oxidative stress and mediate the effects of psychological stress on the skin and hair follicles (Matarrese et al, 2024; O’Sullivan et al, 2022). Both factors are implicated as potential triggers of disease onset in AA and vitiligo, in part through effects on apoptotic and cell regulatory pathways. Notably, the acute stress mediators norepinephrine and dopamine have been shown to influence the hair cycle, and a burst release of norepinephrine was shown to lead to melanocyte stem cell depletion within hair follicles (O’Sullivan et al, 2022; Rachmin et al, 2021).

A second notable cluster of annotations involved SNARE complex assembly and negative regulation of exocytosis, highlighting potential dysregulation in vesicle-mediated melanosome transfer from melanocytes to keratinocytes, the critical final step in skin and hair pigmentation. Two AA_vitiligo common DEGs, namely *STXBP6* and *SNCA* (the latter also identified as a hub gene), encoding for syntaxin binding protein 6 and alpha-synuclein, respectively, are directly involved in this process. In detail, STXBP6 is a negative regulator of SNARE complex assembly and thus vesicle trafficking, whereas the knockdown of *SNCA* in human epidermal melanocytes has recently been shown to impact melanocyte morphology and result in decreased transfer of melanosomes to keratinocytes in cocultures (Rachinger et al, 2022). Further supporting the relevance of pigmentation pathways, the AA_vitiligo common DEGs were enriched for human phenotype ontologies related to hair color and pigmentation, and several other hub genes, namely *MITF*, *DCT*, *LEF1*, and *SLC45A2*, all of which are known mediators of melanocyte biology, have been associated with the hair color trait by GWASs (GWAS catalog, https://www.ebi.ac.uk/gwas/). Taken together, these findings support the previously postulated etiological link between AA and pigmentation traits (Messenger and Bleehen, 1984; Ortonne and Jeune, 1978; Xie et al, 2022; Yousaf et al, 2021) and suggest that shared dysregulation of pigmentation-related processes in epidermal and follicular melanocytes and/or keratinocytes may represent a mechanistic basis for comorbid AA and vitiligo development.

We also made an interesting observation that *SOX10* emerged as the top hub gene for AA_vitiligo pair interacting directly with 5 other hub genes (Figure 3b). SOX10 is a key TF in the regulation of melanocyte development and differentiation. A previous study (Hedstrand et al, 2001) identified SOX10 and SOX9 as possible autoantigens driving vitiligo development in autoimmune polyendocrine syndrome type I. This monogenic disorder is caused by *AIRE* variants and involves organ-specific (poly)autoimmunity, whereby ∼30% of patients develop AA (Collins et al, 2006). The authors had shown that of the 19 patients with autoimmune polyendocrine syndrome type I with vitiligo, 12 (63%) had comorbid AA, and 9 (75%) of these had SOX10 antibody-positive serum in their study. Notably, serum reactivity to both SOX9 and SOX10 correlated with comorbid AA_vitiligo and not with AA alone (Hedstrand et al, 2001). Therefore, we hypothesize that comorbid AA_vitiligo may—at least in a subgroup of patients—have a classical autoimmune etiology, whereby SOX9 and/or SOX10 are shared autoantigens targeted both in epidermal and follicular melanocytes. This hypothesis could align by a previously published compelling histochemical image showing direct interaction between a CD8^+^ T lymphocyte and a follicular melanocyte within a lesional AA hair follicle (Bertolini et al, 2017) and warrants further exploration through mechanistic studies. Finally, another interesting observation along this line was the emergence of STAT3 as a critical regulatory node in the TF–hub gene interaction network. Heterozygous gain-of-function variants in *STAT3* are known to cause multisystem autoimmunity, affecting multiple organ systems (Flanagan et al, 2014), and we observed intriguingly that STAT3 directly controls the expression of 6 AA_vitiligo hub genes in our study.

The common DEGs identified for AA_PSO were significantly enriched in 4 annotations, among which IL-27–mediated signaling was particularly intriguing. Depending on the context, IL-27 can act as either a pro or anti-inflammatory cytokine (Xu et al, 2024), and it has not previously been linked to AA pathobiology. Notably, IL-27 promotes IFN-γ production and supports the T helper (Th)1 immune response, a well-established driver of AA. At the same time, it suppresses the Th17 immune axis (Xu et al, 2024), which is central to PSO pathogenesis. This suggests that dysregulation of IL-27 signaling may underlie comorbidity between AA and a specific Th1-dominant subtype of PSO rather than the typical Th17-driven form. Alternatively, IL-27–mediated shifts between Th1 and Th17 responses could contribute to the Renbök phenomenon, a clinical observation in which one disease inhibits the development of the other within the same anatomical site. In addition, we observed that *CASP1*, a core component of the NLRP3 inflammasome, and *IL1β*, a key downstream effector of inflammasome activation, were among the AA_PSO hub genes (Fetter et al, 2023). This finding raises the possibility that inflammasome activation may play a role in the comorbid development of AA and PSO, warranting further investigation through mechanistic studies.

The common DEGs identified in AA_AD demonstrated significant enrichment in pathways and biological processes associated with both the immune response and skin biology. Among the hub genes, *MX1*, which plays a role in early antiviral defense, and *C1QB*, a component of the classical complement pathway, point to the involvement of the innate immune system. At the same time, components of the adaptive immune response are also implicated through the identification of *HLA-DRA* and *HLA-DPA1* (major histocompatibility complex class II molecules involved in antigen presentation) as well as *LCK*, encoding a tyrosine kinase essential for TCR signaling. Notably, only 1 hub gene, namely *CCL18*, was directly linked to the Th2 immune axis, which is classically associated with AD. Instead, the presence of *PSMB9*, *IRF1*, *MX1*, *CXCL9*, and the 2 *HLA-D* genes suggests an IFN-γ–dominated immune milieu consistent with an Th1-biased immune activation in this comorbid profile. This observation aligns with prior insights into Th1 involvement in specific AD subtypes. In particular, Kageyama et al (2021) demonstrated that the immune profile in patients with comorbid AA and AD differs by AD subtype—with intrinsic AD, characterized by normal IgE levels and less barrier dysfunction, showing a tendency toward Th1-dominated responses, in contrast to the Th2 predominance typically seen in extrinsic AD. Thus, it is plausible that our transcriptomic data analysis results reflect a specific comorbid profile of AA with intrinsic-type AD, marked by a stronger Th1 immune signature.

This study is subject to 2 major limitations. First, the incomplete and nonuniform availability of clinical metadata (including age, sex, disease severity, and ethnic background) across the analyzed datasets has constrained the systematic assessment of potential confounding variables and may affect the generalizability of the findings across diverse populations. Second, the absence of lesional skin biopsy samples from comorbid patient groups and from appropriately matched healthy controls precluded experimental validation of the identified hub genes and pathways. Addressing these limitations through prospective studies using well-phenotyped cohorts will be essential for future mechanistic investigations. Despite these limitations, the conservative analytical framework we applied has demonstrated that comorbidity between AA and other CISDs is noncoincidental and involves overlapping pathobiological processes. Although comorbid AA_vitiligo development may be more attributable to intrinsic skin/hair follicle factors than dysregulation of the systemic immune response, both are implicated in the comorbidity between AA and AD or PSO. Our findings provide a foundation for a more refined classification of AA subtypes based on its common comorbid presentations with distinct CISDs and offer direction for future mechanistic investigations.

## Materials and Methods

### Microarray data and identification of gene expression signatures

Case-control, microarray-based transcriptome datasets for AA (GSE68801), vitiligo (GSE65127), PSO (GSE63741; own data JW), and AD (GSE63741; own data JW) were retrieved from Gene Expression Omnibus (https://www.ncbi.nlm.nih.gov/geo/) (Table 1). Original studies were conducted respectively in the United States, France, and Germany, and all reported that skin biopsies were obtained from lesional skin of patients with clinically active disease at the time of sampling. To identify gene signatures (ie, DEGs) specific to each phenotype, the datasets were first analyzed individually using the limma package in R. The AA dataset was generated in 6 microarray batches; therefore, microarray batch was included as a covariate in the differential expression analysis. No batch structure was reported for the remaining datasets. DEGs were defined using an adjusted *P* < .05 and |log fold change| ≥ 0.58 (Figure 1). Cross-phenotype DEG overlaps, that is, common DEGs, were determined (Figure 2a) and evaluated for statistically significant enrichment using a 1-sided Fisher’s exact test (Figure 2b and Supplementary Table S1). Enrichment *P*-values were corrected for multiple testing using the Benjamini–Hochberg procedure, applied jointly across all pairwise comparisons (n = 6), and are reported as false discovery rates (Figure 2b and Supplementary Table S1). To account for differences in microarray gene coverage, cross-pair enrichment analyses were performed using comparison-specific background gene sets. The GSE63741 dataset was generated using the PIQOR Skin 2.0 Microarray, which targets a predefined subset of transcripts (n = 1557), whereas GSE68801 and GSE65127 were generated using a common microarray platform with broader gene coverage (n = 22,834). For each cross-disorder comparison, background genes were defined as the intersection of genes assayed in both microarray platforms, thereby restricting enrichment analyses to genes measurable in both datasets.

**Table 1:** Transcriptome Datasets from Patients with AA, Vitiligo, PSO, and AD and their Respective Healthy Controls

Disease/Tissue	Number of Samples (n = Case/Control)	Dataset	Source
AA/scalp biopsies	37/23	GSE68801	Jabbari et al., 2016
Vitiligo/skin biopsies	10/10	GSE65127	Regazzetti et al., 2015
PSO/skin biopsies	30/30	GSE63741	D’Erme et al., 2015
AD/skin biopsies	30/30	GSE63741	D’Erme et al., 2015

Abbreviations: AA, alopecia areata; AD, atopic dermatitis; PSO, psoriasis.

### Gene ontology and functional enrichment analysis

For both common and individual DEGs, Reactome pathway enrichment, gene ontology, and human phenotype ontology analyses were performed using Enrichr web tools (http://amp.pharm.mssm.edu/Enrichr/). For gene ontology and functional enrichment analyses of common DEGs across disease pairs, the background gene set was defined as the intersection of genes assayed in the 2 datasets under comparison. For enrichment analyses of individual (disease-specific) DEGs, the background gene set consisted of the full set of genes assayed on the respective microarray platform. Enrichment *P*-values were corrected for multiple testing using the Benjamini–Hochberg procedure, applied jointly across all annotation databases analyzed.

### PPI network construction and analysis

On the gene level, the association between AA and each of the selected comorbid CISD was further investigated by examining the PPI networks of the respective common DEGs. PPI networks were constructed in STRING (https://string-db.org/) and were analyzed and visualized using the cytoHubba (Chin et al, 2014) plug-in of Cytoscape 3.10.1. For each constellation (ie, AA_vitiligo, AA_AD, and AA_PSO), the top 10 hub nodes were identified using degree centrality. Ties were resolved using maximal clique centrality; for AA_vitiligo, where maximal clique centrality was nondiscriminatory, and edge percolated component was applied as an additional criterion.

### Identification of TFs and microRNAs regulating hub genes

Hub genes retrieved from the PPI networks were used as input to construct TF–hub genes and microRNA–hub genes regulatory networks depicting the major regulatory elements of hub gene expression. Here, ChEA3 in NetworkAnalyst 3.0 (Xia et al, 2014) was utilized to identify TF–hub gene interactions, and TarBase (version 9.0) was employed to retrieve microRNA–hub gene interactions. After constructing the networks, network analyses were performed to determine core TFs and microRNAs using a degree-based method.

## Ethics Statement

This study exclusively analyzed publicly available gene expression data from the Gene Expression Omnibus database (GSE68801, GSE65127, and GSE63741) that do not contain personal identifiers. Ethical approval and written informed consent for the original sample collection were obtained by the respective investigators, as documented in the corresponding primary publications. The secondary analyses performed in this study did not require additional ethical approval or written informed consent.

## Data Availability Statement

The datasets used in this study (https://www.ncbi.nlm.nih.gov/geo/query/acc.cgi?acc=GSE68801, https://www.ncbi.nlm.nih.gov/geo/query/acc.cgi?acc=GSE65127, and https://www.ncbi.nlm.nih.gov/geo/query/acc.cgi?acc=GSE63741) were downloaded from the Gene Expression Omnibus database (https://www.ncbi.nlm.nih.gov/geo/).

## ORCIDs

Hadis Abdolahzadeh: http://orcid.org/0000-0001-7451-0909

Sabrina Henne: http://orcid.org/0000-0002-2953-2700

Amir Mokhlesi: http://orcid.org/0009-0004-4320-8837

Jörg Wenzel: http://orcid.org/0000-0002-4744-5993

Stefanie Heilmann-Heimbach: http://orcid.org/0000-0003-1057-465X

Regina C. Betz: http://orcid.org/0000-0001-5024-3623

F. Buket Basmanav: http://orcid.org/0000-0001-6411-4275

## Conflict of Interest

The authors state no conflict of interest.
